# Charity

**Published:** 1903-01

**Authors:** F. E. Daniel


F. E. DANIEL.

### CHARITY.

### Address at the Elks' Memorial, Austin, Texas, December 7, 1902.

F. E. DANIEL.

"Charity" is perhaps the broadest and most comprehensive word in the English language. It is all-embracing. Under its broad signification are included the highest virtues and the noblest sentiments of the human heart. It is the mother of Mercy, and twin-sister to Faith and Hope. It is the theme of the poet in all ages; the ideal of the painter and the sculptor.

The word is derived from the Latin *caritas,* which means "dearness," "high regard," "love;" from *carus,* "dear," "costly." Its equivalent and synonym in the Sanscrit is *kam,* "to wish," "to love." In the Irish tongue *cara,* "a friend." In the Welsh, *caru,* *"to love."* Thus it will be seen to embrace: Love, Benevolence, Friendship, Good-will; Affection, Tenderness; Beneficence; Liberality, Mercy, Pity. It is the mountain and source whence flows that sympathy, a touch of which makes all the world akin;—whence spring the gentlest and best emotions of the heart; pity for the unfortunate—mercy for the erring. It ever puts the best possible construction upon the motives, acts and deeds of others.

Its "quality," like that of mercy, "is not strained. It falleth as the gentle rain from heaven on the place beneath. It is twice blest; is blesseth him that gives and him that takes. * * * It becomes the throned monarch better than his crown. * * * It is an attribute to God himself." It fall upon the broken and bruised heart like the dews of heaven upon the drooping flower. It blesses, feeds and cheers. Charity is the pabulum of the soul. God has implanted its germ in every heart. Some cherish it, some smother and stifle it; but with its cultivation and exercise every true soul grows and expands with good deeds and ripens for immortality.

Almsgiving, alone, is not charity. It is but a narrow phase of the divine attribute. The mere relief of the physical needs of the unfortunate and less favored is sometimes grudgingly done, or done for the glorification of the giver. True charity is the impulse, the feeling, the love of one's kind, and pity and sympathy which prompt the gift. For "tho' I bestow all my goods to feed the poor, * * * and have not charity, it profiteth me nothing."

"Life is not that which, without us we find,
Chance, accident, merely, but rather the mind,
And the soul that within us surviveth these things;"

And

"Our real existence hath truly its springs
Less in that which we do than in that which we feel."

"He most lives who thinks most, feels the noblest, acts the best."

Practical charity is the outward expression of this God-given instinct. It is manifested in the founding and operation of those great benefices of the world,—the hospitals and sanitaria,—the asylums, wherein the broken, the maimed, the blind, the afflicted are tenderly cared for, and the dying are comforted and consoled; the schools in which the benighted minds of the children of the poor are illuminated and enlightened by learning; the missions, through whose ministrations the erring and illiterate are pointed the way to heaven; to Him,—the Source and Center of all charity, all benevolence,—all love.

Charity is the heaven-born inspiration that animates and actuates that noble and devoted band of benefactors, the Sisters and the Nuns,—who, suppressing all earthly hopes and desires, and eliminating self, dedicate themselves to deeds of mercy and practical charity, sustained by faith, and cheered by the hope of a glorious reward beyond the skies. See the gentle Sister, in sombre garb and snowy cap, serenely walk into the valley of death! She walks the wards of the pest-plagued hospital, with silent and gentle step, and a saint-like smile that illumines her whole being, like the halo we see in holy pictures. Around the sin-seared soul she wraps the mantle of charity. She pours the balm of human sympathy into the bleeding and bruised heart. She lights the torch of hope in the bosom of despair. She blesses and soothes, and makes easy the flight of the departing spirit, and with prayer and benediction speeds it upon the wings of peace to the bosom of God. And when she herself is stricken, as she knows she must be, she bravely perishes, as she has lived, without protest or plaint, and cheerfully gives her life for charity's sake. She is sure of the reward of the pure in spirit; they shall see God.

And the noble order of the Red Cross. What sends these devoted disciples of the Divine Healer across the seas and desert sands to the battle-fields and the hospitals, where lie broken and bleeding, and often torn and trampled out of all semblance of human kind, those who fall in the fray? What sends them to the famine-stricken people in foreign lands, with stores of comfort and relief? The divine impulse, Charity, the mother of Mercy, the mother of Pity,—of Love. God bless them. All honor to the Nuns! All honor to the Red Cross ministers of mercy!

Buckminster said: "The highest exercise of charity is charity toward the uncharitable." This is but an echo of the sentiment so oft repeated in the Scriptures: "Love thy neighbor as thyself;" "Do good to them that despitefully use you." It is an echo of the conception of true charity that has sounded down the age since Pittacus of Mithylene, one of the Seven Wise Men of Greece, said, six hundred and fifty years before Christ: "Do not that to thy neighbor that thou wouldst take ill at thy neighbor's hands." It was echoed one hundred and fifty years later by Confucius,—five hundred years before Christ said: "Do unto others as you would that others should do unto you." The gentle Nazarene taught this charity toward the uncharitable not only by precept, but by the most illustrious example ever recorded in the annals of time. When the Savior of man left the courts of heaven to redeem a fallen race, with a heart full of love and pity,—pregnant with divine Charity, he became an object-lesson in "charity toward the uncharitable," the most aweful,—the most sublime. When the crest and crown of Calvary were made forever glorious by the enactment of the Divine Tragedy; when "suns and stars and heaven and earth rocked and reeled to the martyrdom of a god,"—before the gentle spirit, cruelly torn from the frail and broken body, winged its flight to the Eternal,—he lifted up his beautiful face and cried: "Father,—forgive them; they know not what they do." A wondering world never witnessed aught so grand, so sublime, so awe-inspiring; and for two thousand years this example has been the beacon light to guide and lead to the better way. Enshrined in the hearts of man he is deified and worshiped,—the very apotheosis of all that is pure and perfect,—of Love, of Mercy, of Good-will, —of Charity.

The Benevolent and Protective Order of Elks have caught the inspiration, and they practice charity. It is one of the cardinal principles and purposes of the order. They do good by stealth, letting not the left hand know what the right hand doeth. Around the world this noble organization is sending out its tendrils and branches, and with the "hailing sign" brother greets brother across the seas. It takes root and grows, wherever true men and good are found; and the West and the East, and the North and the South, are being united in its fraternal bonds. With limited means, such an organization cannot accomplish the great practical charities by which men judge, but they teach by precept and example. They do good silently, like the dews of heaven, in the stillness of the night. Living, they are held in the bonds of brotherly love. Dying, they live in our memories. "With charity we write their faults upon the sands. Upon the tablets of our memory, with love, we inscribe their virtues."

Our departed brothers; where are they? Shall we see them again? A young Roman patrician in the days of Cæsar was condemned to death for some political sin. He was permitted to take leave of his betrothed. With her arms entwined around his neck in fond and final embrace,—gazing into his eyes she asked: "Shall we ever meet again?" He replied: "I have asked that question of the skies. I have asked it of the stars. I have asked it of the eternal spheres that ceaselessly roll through the boundless space of the universe,—instinct with Deity,—vibrant with life and love, in harmony with and obedient to the will of Him who made it. But the question falls back unanswered, upon an aching and anxious heart. But when I gaze into the fathomless depths of your beautiful eyes, and see there a soul so pure, so angelic,—so fitted for celestial scenes, I am answered: We *shall* meet again! Oh, my beloved,—meet me there!"

Our departed brothers. We meet; we miss them. "Oh for a touch of the vanished hand,—a sound of the voice that is still!" Shall we see them again? From the depths of the heart this question ever rises to the lips. Who can answer it? "It must be so. Plato, thou reasonest well. Else why this pleasing hope,—this fond desire,—this longing for immortality?" The voice of Reason says: "No. It cannot be." But the eye of Faith catches the glint and gleam of celestial robes, and the ear of Hope hears the rustling of angel wings. If we cannot know, we can believe,—we can hope. We *shall* see them again! Hand shall grasp hand and heart shall speak to heart! When the Exalted Ruler of the Universe shall assemble the Elks of the world in the grand and final conclave beyond the shores of time,—in that abode of perfect peace and rest where abides the Great First Cause; where Charity, all pervading, rules supreme and throws her celestial mantle over all faults and failings; where Mercy blots out all sin, and love and sympathy soothe all sorrows,—to the roll-call of Austin Lodge No. 201 all our brothers who have gone before,—the gentle and lovable Patrick,—the cheery, dear, delightful Morse, the joyous young Goldbeck,—the bright and beautiful O'Connor will answer: "I-am-here."

